# Successful treatment of acute worsening complex regional pain syndrome in affected dominant right-hand from secondary pathology of new onset third and fourth digit trigger finger

**DOI:** 10.1080/23320885.2022.2063871

**Published:** 2022-04-27

**Authors:** Monika Patel, Michael Aiello

**Affiliations:** a Department of Anesthesiology, Division of Pain Management, University of Florida College of Medicine, Jacksonville, FL, USA; b Department of Anesthesiology, University of Florida College of Medicine, Jacksonville, FL, USA

**Keywords:** Complex regional pain syndrome, CRPS, trigger finger, stenosing tenosynovitis, tendon sheath injection

## Abstract

65 year old male with preexisting Complex Regional Pain Syndrome (CRPS) in right dominant hand with sudden onset of right third and fourth digit trigger finger successfully treated with flexor tendon sheath corticosteroid and lidocaine injection resulting in long-term resolution of symptoms without causing widely believed aggravation of CRPS.

## Introduction

Complex Regional Pain Syndrome (CRPS) typically affects a single extremity causing pain and debility that can have serious physical and psychological impacts on quality of life. Although rare, it can arise after minor trauma such as needle stick injury or after a surgical intervention such as carpal tunnel syndrome [1]. In the acute phase there is an increase in pro-inflammatory cytokines leading to swelling, redness, connective tissue contractures, and high turnover osteoporosis [2]. Also, an increase in neuropeptides leads to sympathetic hyperactivity of the affected limb causing skin temperature and color changes [2]. In chronic CRPS sustained symptoms are hypothesized to be due to neuroplasticity of the central nervous system with possible genetic predisposition, limb disuse, or psychosocial risk factors [2].

Diagnosis can be established by using the Budapest Criteria [3]. Radiographic imaging with a 3-phase bone scan, MRI and plain X-ray may also assist in confirming the diagnosis and ruling out other conditions [2]. Beneficial treatments include non-steroidal anti-inflammatory medications, neuropathic agents, bracing, physical therapy, occupational therapy, sympathetic block intervention, spinal cord stimulation, dorsal root ganglion stimulation, and scrambler therapy on the affected limb [4,5]. Invasive procedures and surgery on the extremity associated with CRPS are generally avoided due to an increased risk of worsening CRPS symptoms or reoccurrence [6]. Occasionally, the patient may develop additional pathologies in the CRPS affected extremity limiting treatment options due to concerns of aggravating CRPS symptoms.

### Purpose

This is a case of acute worsening CRPS due to sudden onset of right third and fourth digit trigger finger causing severe pain and limited digit range of motion treated with flexor tendon sheath injection resulting in long-term resolution of symptoms without causing widely believed aggravation of CRPS. Patient consent was received to publish this case report.

### Case

A 65 year old right-hand dominant male with a past medical history significant for diabetes, lupus, arrhythmia on life-long anticoagulation, and extensive cervical to lumbar spinal fusion surgeries presented with acute onset right distal arm CRPS type 1 diagnosed by Budapest Criteria [3]. Patient-reported severe pain in the right forearm and hand not following a dermatomal pattern that occurred about 1 month after undergoing right carpal tunnel release surgery. Physical exam findings revealed sensory changes of allodynia over the right palm, palmar wrist, and portions of the dorsal hand and hyperalgesia over the distal right forearm; vasomotor changes of cooler right distal fingers, erythema of the hand, bluish discoloration of the wrist; sudomotor changes of right-hand swelling and excessive palmar sweating; and motor/trophic changes of right-hand grip strength weakness, loss of finger flexion range of motion to 90 degrees, limited right wrist flexion to a few degrees, and shiny skin changes without nail changes.

Multimodal treatments included occupational therapy for improved range of motion, hand and finger bracing, desensitization techniques; two sessions of scrambler therapy about 1 year apart; and neuropathic and opioid pain medications. Over the subsequent years, patient’s symptoms improved to a tolerable level of right-hand pain and improved finger range of motion. However, he suffered from continued allodynia and discoloration of the right wrist and palm, sudden shooting pains in the hand, and frequent dropping objects from the hand. He continued to take nortriptyline 60 mg daily, oxycodone/acetaminophen 5/325 mg twice a day as needed, and duloxetine 60 mg daily. Sympathetic block or spinal cord stimulation interventions were not pursued due to life-long anticoagulation and previous extensive cervical to lumbar spine fusion surgeries.

### Trigger finger

After three years of having CRPS symptoms, he developed acute worsening of right-hand pain and was unable to extend the right third and fourth digit. Physical exam revealed the onset of trigger finger of the right third and fourth digit with painful finger extension and loss of finger range of motion. Typically, trigger finger also called stenosing tenosynovitis is caused by hypertrophy of the finger flexor tendon sheath A1 pulley resulting in digit locking in a flexed position [7]. Pain was excruciating and severely worse than the previous baseline CRPS symptoms. The pain was constant in nature, impairing sleep, and causing the patient to become severely depressed with suicidal ideation. Additionally, activities of daily living were becoming increasingly difficult due to the loss of right-hand function.

Treatment with occupation therapy, finger splinting, topical anti-inflammatory treatments, and an increase in opioid medications did not improve pain. After discussing the risk of possibly worsening CRPS versus the possible benefit of performing a trigger finger injection into the flexor tendon sheath with corticosteroid and anesthetic, the patient agreed to proceed with the injection [8]. Treatment solution containing 0.5 mL of preservative-free lidocaine 1% and Triamcinolone 20 mg was injected at the right third flexor tendon sheath near the palmar crease at the point of maximal tenderness using a 30 gauge 1.6 cm needle. The patient tolerated the procedure well and noticed an immediate improvement in pain.

## Results

The patient reported considerable improvement in the right third digit stiffness and locking about two days after the procedure. He reported he could extend the digit, but his right fourth digit was still flexed. The procedure was subsequently performed at the right fourth digit about 5 weeks later. There was an improvement in right-hand pain, resolution of third and fourth trigger finger locking, and improved depressive symptoms that continued long-term.

At the follow-up visit, the patient reported the CRPS symptoms were essentially resolved with no pain, return of normal skin coloration, temperature, and sensation in the right hand. He reported complete functional return of the right hand, including writing, except for opening jars which were limited by handgrip strength. He did notice the slight return of only the third digit trigger finger about 18 months after the first injection. Flexor tendon sheath injection was repeated at the third digit with corticosteroid and anesthetic with a resolution of pain. The patient was able to wean off neuropathic and opioid pain medication due to improvement in symptoms. He did decide to continue taking nortriptyline and duloxetine for his mood.

## Discussion

The presented case shows the dynamism of CRPS symptoms benefiting from long-term assessment and identification of treatable superimposed conditions such as trigger finger. Despite the risk of worsening complex regional pain syndrome, flexor tendon sheath injection with corticosteroid and lidocaine provided significant benefit. The patient had improved pain, increased finger range of motion, improved fine motor skills of the dominant right hand including writing, holding objects, sleeping at night, and hygiene. The patient did not need to continue hand bracing or opioid medications.

The ongoing assessment of chronic CRPS provided valuable identification of superimposed trigger finger diagnosis and long-term outcome. Although it is unclear if the CRPS resolution after tendon sheath injection is causal or coincidental, the remarkable recovery is noteworthy. Treatment of CRPS using corticosteroids and lidocaine has been thought to reduce the sympathetic response, c-fiber activation, inflammation, and pain [9]. Consider evaluating for stenosing tenosynovitis diagnosis in acute finger range of motion loss with a sudden worsening complex regional pain syndrome of the hand. Additionally, consider treatment with corticosteroid and lidocaine injection of the flexor tendon sheath, which provided long-term benefit in the presented case.
